# Lupus Nephritis from Pathogenesis to New Therapies: An Update

**DOI:** 10.3390/ijms25168981

**Published:** 2024-08-18

**Authors:** Annalisa Roveta, Emanuele Luigi Parodi, Brigida Brezzi, Francesca Tunesi, Valentina Zanetti, Guido Merlotti, Alessia Francese, Antonio G. Maconi, Marco Quaglia

**Affiliations:** 1 Research and Innovation Department (DAIRI), “SS Antonio e Biagio e Cesare Arrigo” University Hospital, 15121 Alessandria, Italy; aroveta@ospedale.al.it (A.R.); alessia.francese@ospedale.al.it (A.F.); amaconi@ospedale.al.it (A.G.M.); 2 Nephrology and Dialysis Unit, “SS Antonio e Biagio e Cesare Arrigo” University Hospital, 15121 Alessandria, Italy; emanuele.parodi@ospedale.al.it (E.L.P.); bbrezzi@ospedale.al.it (B.B.); 3 Nephrology and Dialysis Unit, IRCCS “San Raffaele” Scientific Institute, 20132 Milan, Italy; tunesi.francesca@hsr.it; 4 Department of Internal Medicine, University of Genova, 16126 Genoa, Italy; valentinazanetti94@gmail.com; 5 Department of Primary Care, Azienda Socio Sanitaria Territoriale (ASST) of Pavia, 27100 Pavia, Italy; gmerlotti87@gmail.com; 6 Department of Translational Medicine, University of Piemonte Orientale (UPO), 28100 Novara, Italy

**Keywords:** systemic lupus erythematosus (SLE), lupus nephritis (LN), pathogenesis, immunosuppressive (IS) therapy, renal biopsy, biomarker, belimumab (BEL), voclosporin (VOC), chronic kidney disease (CKD), end-stage renal disease (ESRD)

## Abstract

Lupus Nephritis (LN) still represents one of the most severe complications of Systemic Lupus Erythematosus (SLE) and a major risk factor for morbidity and mortality. However, over the last few years, several studies have paved the way for a deeper understanding of its pathogenetic mechanisms and more targeted treatments. This review aims to provide a comprehensive update on progress on several key aspects in this setting: pathogenetic mechanisms of LN, including new insight into the role of autoantibodies, complement, vitamin D deficiency, and interaction between infiltrating immune cells and kidney resident ones; the evolving role of renal biopsy and biomarkers, which may integrate information from renal histology; newly approved drugs such as voclosporin (VOC) and belimumab (BEL), allowing a more articulate strategy for induction therapy, and other promising phase III-immunosuppressive (IS) agents in the pipeline. Several adjunctive treatments aimed at reducing cardiovascular risk and progression of chronic renal damage, such as antiproteinuric agents, represent an important complement to IS therapy. Furthermore, non-pharmacological measures concerning general lifestyle and diet should also be adopted when managing LN. Integrating these therapeutic areas requires an effort towards a holistic and multidisciplinary approach. At the same time, the availability of an increasingly wider armamentarium may translate into improvements in patient’s renal outcomes over the next decades.

## 1. Introduction

Lupus Nephritis (LN) represents one of the most severe and frequent complications of Systemic Lupus Erythematosus (SLE) and a major risk factor for morbidity and mortality, potentially leading to end-stage renal disease (ESRD) [1].

Etiopathogenetic mechanisms are complex and involve multiple inflammatory pathways and cell types, far exceeding immune complex (IC) deposition. Significant progress has been made over the last years in understanding the role of innate immunity cells, such as neutrophils, monocytes and dendritic cells (DC) [2] and the interaction between them and kidney resident cells [3]. At the same time, the increasing availability of new serum and urinary biomarkers is changing the role of renal biopsy as a diagnostic and prognostic tool [4]; newly approved drugs, such as voclosporin (VOC) and belimumab (BEL), are allowing a more articulate strategy for induction therapy and are even redefining traditional views of induction and maintenance treatment; several other promising phase III-IS agents are in the pipeline are likely to expand the therapeutic landscape in the near future [5]. This review aims to provide a comprehensive update on recent progress on each of these key aspects of LN, with a focus on papers published over the last 4 years (January 2020–June 2024). A combination of Medical Subject Headings (MeSH) and keywords related to SLE, LN, pathogenesis, new therapies, and biomarkers were employed and references of relevant articles were also checked.

## 2. Epidemiology

SLE affects people of all races, genders, and ages. However, it is more commonly observed in high-income nations, particularly among women in their thirties to forties, with a female-to-male ratio of around 9:1. The worldwide incidence of SLE is estimated to be 5.14 per 100,000 person-years and the prevalence would be 43.7 per 100,000 individuals, affecting a total of 3.41 million people. However, epidemiological data on SLE are missing in 79.8% of nations [6]. Overall more than 10% of kidney biopsies lead to a diagnosis of LN, which affects around 40% of SLE patients [7,8,9], representing the most frequent secondary glomerular disease [10,11,12,13]. In around one-third of patients, it is the presenting feature leading to the diagnosis of SLE [14].

The prevalence of SLE and the chances of developing LN vary considerably between different regions of the world, socioeconomic status, and ethnicities [15]. A greater prevalence of LN is documented among individuals of Hispanic, African, and Asian descent when compared to Caucasians [16,17,18,19]. The prevalence of LN within a total number of diagnoses in renal biopsy registries of different countries is outlined in Table 1.

The overall incidence of LN seems to be higher in women than men, with a male-to-female ratio of approximately 1:5 [13,18,30]. Women had incidence rates up to 10 times greater than men in the 30–39 year age group, which decreased to levels comparable to those of men after the age of 60, according to a Danish Registry study [31].

Despite progress, LN prognosis remains rather unpredictable and around 10–30% of patients progress to ESRD within the first ten years of the disease [13].

Patients of Hispanic and African descent are characterized by increased disease activity and relapse rates, more rapid progression to chronic kidney disease (CKD) and early mortality [13,16,32], whereas male sex and increased creatinine levels at diagnosis are independent prognostic elements for CKD in Caucasians with LN.

The incidence of ESRD in wealthy nations experienced a significant decline from the 1970s to the mid-1990s and it has since stabilized [33], whereas poverty is a significant risk factor for progression of LN, regardless of race or ethnicity [34].

## 3. Pathogenesis of Lupus Nephritis (LN)

The pathogenesis of LN is complex and multi-factorial. It involves a variety of extra- and intra-renal pathogenic mechanisms, resulting from genetic predisposition as well as environmental and hormonal factors [35]. We will focus on the most important progress in understanding LN pathogenetic mechanisms.

### 3.1. Genetics and Epigenetics

Over 100 susceptibility loci in the human genome are linked to SLE and LN. Genetic variants are involved in loss of tolerance against nuclear autoantigens, abnormal lymphocyte and complement function and kidney damage. Genetic variants contribute to the racial disparities and clinical heterogeneity of SLE and LN [36].

The strongest LN genetic association relates to the major histocompatibility complex (MHC) region, with an increased risk due to amplified tissue inflammation (*HLA-DR2* and *HLA-DR15*); on the other hand, different HLA alleles may exert a protective effect (*HLA-DR4* and *HLA-DR11*) [36]. An association between the burden of SLE risk loci and the risk of LN was found in two large multi-ethnic cohorts of 1237 SLE patients. Genetic risk score effects of HLA and non-HLA loci increased in magnitude when analysis was restricted to proliferative classes [37]. Zhang et al. screened a Chinese cohort of 1886 patients with LN by whole-exome sequencing and identified only a small fraction of patients with pathogenic gene variants, primarily related to NF-kB, type I interferon (IFN-I), PI3K/AKT, JAK/STAT, RAS/MAPK and complement pathways [38].

A genome-wide association study of LN in a Han Chinese population found some promising candidates associated with LN, especially polymorphisms related to Transforming Growth Factor-β (TGF β) pathways [39].

Yavuz et al. recently showed that variants in the Mer-tyrosine kinase (*MERTK*) gene modulate the risk of developing ESRD in SLE [40]. Interestingly, MERTK is a member of the Tyro3/Axl/Mer receptor kinase family and the main receptor for apoptotic cells on macrophages, playing a key role in the regulation of efferocytosis [41].

In addition to this complex scenario of modulating polymorphisms, mendelian monogenic defects are involved in causing early-onset LN in children, young adults, male patients, and familial cases. These determine defects in the clearance of apoptotic cells or ICs, interferonopathies, JAK-STAT-TLRopathies, and T and B cell dysregulation [42].

**Figure 1:**
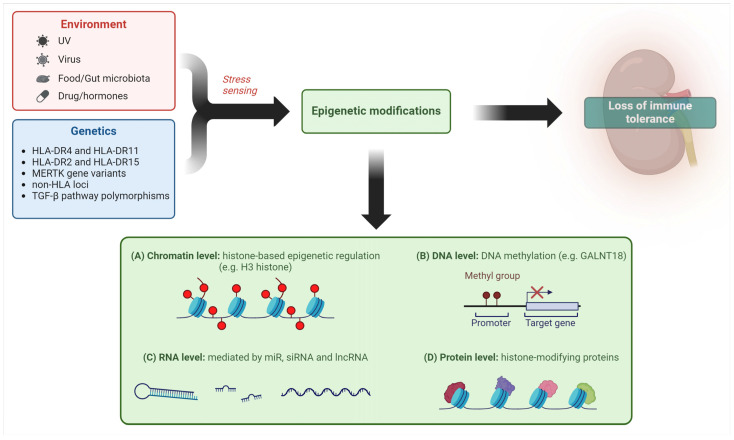
Different epigenetic mechanisms mediate the impact of environmental factors in predisposing to loss of tolerance in LN (created with BioRender.com). Legend. miR—microRNA; siRNA—small interfering RNA; lncRNA—long non-coding RNA.

Mechanisms of epigenetic regulation of gene expression have also been investigated in LN, such as DNA methylation/acetylation and histone and non-histone protein modifications [43], as shown in Figure 1.

Coit et al. demonstrated that demethylation of a CpG site of the *GALNT18* gene in neutrophils was relevant to LN in a 4-year longitudinal analysis [44] and Wu proved that an inhibitor of enhancer of zest homologue 2, an enzymatic subunit of a complex which promotes transcriptional silencing by methylating H3 histone, can mitigate LN in a mouse model [45].

The role of microRNAs (miR) in LN pathogenesis has also been increasingly recognized [46]. Significant progress has been made in defining the actions of non-coding RNA (nc-RNA) as epigenetic regulators of gene expression through mRNA targeting. This family includes long non-coding RNA (lncRNAs), characterized by more extended length and containing circular RNA (circRNA), and miRs. Both lncRNAs and circRNA can increase or blunt the generally inhibitory effect of miRNA. Dysfunction of this complex regulatory system is observed in SLE and LN at different levels, for example in renal resident cells and urinary exosomes, and results in abnormal cell proliferation, inflammation, and fibrosis [47].

### 3.2. Environmental Factors

SLE pathogenesis is probably the result of complex interactions between ethnic, genetic, epigenetic, hormonal and environmental factors, which trigger an immunological disorder. However, only a small number of studies have examined whether environmental exposure interacts with genetic factors, despite the fact that a variety of exogenous triggers, including physical and chemical factors, have been proposed to influence the risk of SLE and LN [48]. We will briefly analyze the most significant ones.

The role of ultraviolet beams (UVB) in modulating the expression of disease is well known [48]. Interestingly, UVB appears to stimulate neutrophil migration also to the kidney in an interleukin (IL)-17A-dependent manner, creating a “kidney-skin” axis [49].

Air pollution has been recently drawing attention. Bai et al. demonstrated that the risk of LN in SLE patients may be increased by short-term exposure to nitrogen dioxide (NO_2_) and fine particulate matter (PM)2.5, but underlying pathogenic mechanisms are still obscure [50].

The role of viral infections in triggering SLE and modulating the risk of LN is well known [51], including recent reports of de novo LN following SARS-CoV-2 infection [52].

An association between dysbiosis and microbial antigens with LN has been emerging. Bacterial metabolites mimicking autoantigens could be a promising target for disease management [53].

Gut microbiota actually appears to modulate renal inflammation, as a “leaky gut” allows pathogenic bacteria to enter the bloodstream and stimulates the formation of antibodies and ICs which deposit in the kidney in experimental models [54].

The role of food in modulating inflammation is an expanding area of research. Supplementation of vitamin D or E and omega-3 fatty acid have been associated with improvement of inflammatory markers and endothelial function in SLE [55] and curcumin appears to exert interesting anti-inflammatory and antioxidant effects [56] along with an anti-proteinuric effect in LN [57]. These data deserve further study but suggest the potential impact of food antigens on autoimmunity.

### 3.3. Immunological Mechanisms

Dysregulation of a wide range of immune system elements characterizes SLE and LN. We will focus on some key aspects of intrarenal immunological mechanisms: the role of pathogenic autoantibodies and the interaction between infiltrating immune cells and resident cells.

#### 3.3.1. Role of Autoantibodies

The role of autoantibodies and IC production in kidney inflammation is well known [58]. Stimulation of autoantibodies requires a source of extracellular DNA in an immunologically accessible form, such as extracellular DNA emerging from dead and dying cells due to abnormal apoptosis [59]. Autoantibodies directed against nuclear and cellular antigens lead to the formation of ICs, which accumulate in both glomeruli and interstitium, activating complement and recruiting immune cells [60].

**Figure 2:**
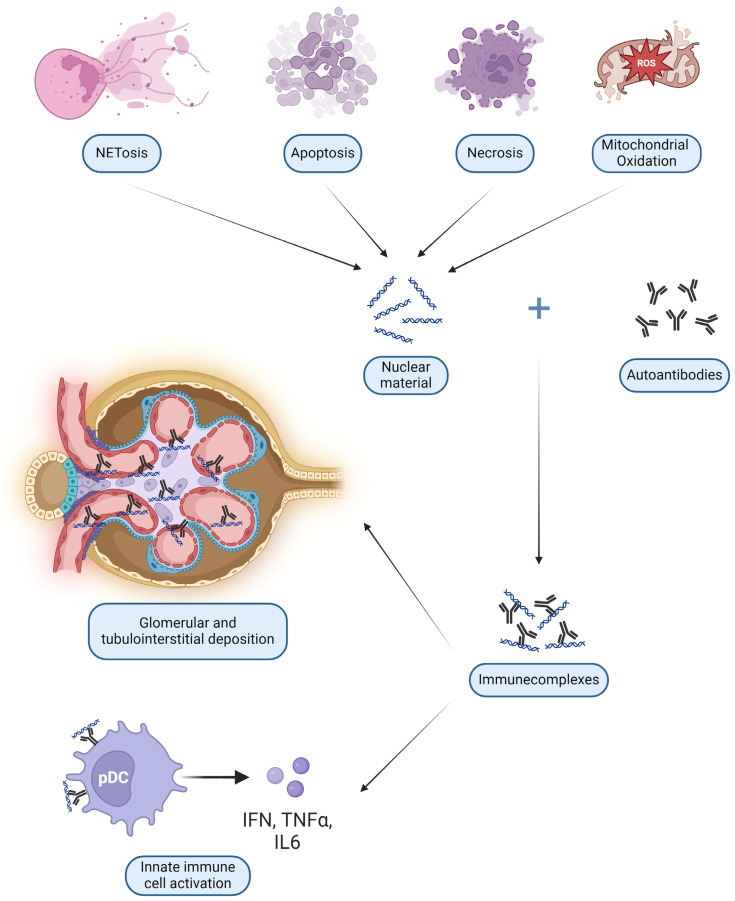
Main mechanisms of damage by nephritogenic immune complexes (IC): deposition in glomeruli and interstitium and direct activation of innate immunity cells such as plasmacytoid dendritic cells (pDC) (created with BioRender.com).

Anti-DNA antibodies are probably the most studied type of autoantibodies. After binding DNA they form ICs with a dual pathogenetic role: glomerular deposition and uptake into innate immune cells. The latter process determines the interaction of nucleic acids with internal sensors, with consequent cell activation and release of cytokines [61]. These DNA and RNA ICs resemble viral particles and elicit the same viral nucleic acid recognition receptors such as Toll-like receptors (TLR) on all antigen-presenting cells (APC), especially B lymphocytes and DC [62]. Plasmacytoid DC (pDC) can then start a broad pseudo-antiviral response, releasing IFN-I and Tumor Necrosis Factor (TNF) α [63], as shown in Figure 2 and detailed in Section 3.3.4.

The specific pathogenetic meaning of traditional types of autoantibodies has been critically reviewed in a recent paper. These Authors underline the weak link between anti-double stranded DNA (anti-dsDNA) and renal pathology and argue that circulating levels of anti-enolase 1 (anti-ENO1) and anti-histone 2 (anti-H2) IgG2 better characterize patients with LN vs. those with non-renal SLE and decrease after IS therapy [60]. Another recently identified autoantibody is represented by anti-superoxide dismutase 2 (SOD2), a ‘second wave antibody’ which may interfere with a protective anti-oxidant mechanism in a late phase of inflammation [64].

Of interest, glycosylation of autoantibodies can modulate their cytotoxic potential in opposite ways, as sialylation reduces [65], whereas fucosylation increases their cytotoxicity [66].

Different autoantibodies co-existing with anti-dsDNA may also contribute to the pathogenesis of LN, including anti-C1q, anti-nucleosome, anti-α actinin, and anticardiolipin (aCL) [60]. Some autoantibody classes such as anti-dsDNA are highly heterogeneous, with multiple target antigens only partly known [67]. Furthermore, considerable cross-reactions exist. For example, α-actinin cross-reacts with anti-dsDNA antibodies and characterizes a subset of anti-dsDNA with high avidity. Interactions with both resident and infiltrating cells are equally complex, as different autoantibodies can trigger a wide range of responses. For example, aCL antibodies can induce mesangial cell (MC) apoptosis [68], whereas anti-dsDNA can stimulate neutrophil extracellular traps (NET) release by neutrophils and increase their immunogenicity [69].

The role of new autoantibodies such as anti-modified/monomeric C-reactive protein (CRP) [70] and anti-NET antibodies (ANETA) [71] is being defined.

#### 3.3.2. The Complex Role of Complement

Complement plays a dual role in LN pathogenesis. Effective clearance of ICs by early complement components protects against the development of LN, as shown by the consequences of genetic defects in complement; however, its uncontrolled activation promotes kidney damage in SLE [72].

While classical pathway activation by ICs was initially regarded as the main contributor to LN pathogenesis, increasing evidence indicates that also alternative and lectin pathways are involved [73].

Excessive complement consumption is a hallmark of SLE. Reduction in plasma levels of C3 and C4 complement fractions indirectly reflects IC formation and immunological activity. C3 reduction, but not C4, correlates with renal flares. These are probably caused by C3 activation in the kidney, promoting inflammation and exposing C3b epitopes with consequent production of anti-complement antibodies, suggesting an important role of alternative pathway [72].

Recent studies have highlighted the role of an elevation of circulating complement split products, C3dg, iC3b, and C4d; interestingly, C3dg/C3 and iC3b/C3 ratios correlate with active SLE and C4d are higher in patients with LN [74].

Cell-bound complement activation products also seem to play a pathogenetic role. For example, erythrocyte-bound C4d levels better correlate with disease activity than low plasma complement levels and are elevated in LN. Moreover, C4d deposition in renal peritubular capillaries predicts a worse prognosis [75].

A better understanding of the contribution of complement pathways to LN pathogenesis may pave the way for novel therapies targeting this system and clinical trials with several anti-complement molecules are ongoing. This approach, however, is complicated by the complement’s dual role, providing protection on the one hand and mediating tissue damage on the other [72].

#### 3.3.3. Vitamin D Deficiency

Vitamin D deficiency (serum 25-hydroxyvitamin D_3_ < 15 ng/mL) is common in SLE due to a variety of reasons: reduced sun exposure to avoid photosensitivity, possible interference by glucocorticoids (GC) and hydroxychloroquine (HCQ), presence of autoantibodies against it. Vitamin D has complex antiproliferative and immunomodulating functions and low levels correlate with consumption of C3 and C4 complement fractions and higher SLEDAI [76].

In a recent study, serum 25-hydroxyvitamin D_3_ levels in patients with initial-onset childhood SLE negatively correlated with T helper (Th)-17 and related cytokines and positively with T regulatory (Treg) subset [77].

Interestingly, patients with LN have lower serum levels than patients with LES without renal involvement and renal tissue expression of vitamin D receptors is reduced and negatively related to the activity index of LN [78]. Expression of vitamin D-synthetizing enzyme 1α-hydroxylase in peripheral blood mononuclear cells is also significantly reduced in SLE patients, especially if LN is present, and negatively correlates to SLEDAI [79].

A growing body of evidence suggests that vitamin D regulates the renin-angiotensin-aldosterone system, inhibits renal inflammation, preserves the expression of nephrin and podocin and protects podocytes damaged by autoantibodies from aberrant autophagy in LN [80].

The activated vitamin D form, calcitriol, can potentiate the antiproteinuric effect of angiotensin 2 inhibitors in LN and other types of GN, also exerting actions on podocytes through the vitamin D receptor [81].

Overall vitamin D deficiency appears to be involved both in the mechanism of immune dysregulation of SLE and in the pathogenesis of LN. Furthermore, it seems to exert a broader nephroprotective effect, as demonstrated also in other nephropathies [81].

#### 3.3.4. Role of Infiltrating Immune Cells

The role of infiltrating immune cells in LN has been the focus of intense research over the last 5 years and will be analyzed separately.

##### Neutrophil

Neutrophils have been emerging as key players in SLE pathogenesis. SLE is characterized by deregulation of hematopoiesis, with inflammatory priming and myeloid skewing of bone marrow-derived hematopoietic stem and progenitor cells (HSPC). Emergency granulopoiesis and extramedullary hemopoiesis (EMH) are amplified and result in an increased release of neutrophils from bone marrow in order to meet the demand for inflamed tissues. EMH develops in the spleen and in the kidneys themselves and correlates with LN severity. Neutrophils produced in these sites are likely involved in endothelial and renal damage [82].

In addition to a strong granulocytic molecular signature in peripheral blood, patients with SLE are characterized by augmented NETosis, a specific form of cellular death in which NETs containing nuclear DNA, chromatin and highly immunogenic and proinflammatory cytoplasmatic proteins are released (Figure 2) [83]. While few intact neutrophils are present in renal biopsy, the persistence of tissue factor and IL 17A-bearing NETs has been demonstrated in proliferative forms of LN and is likely to mediate thrombo-inflammation and fibrosis even in the absence of these cells [84]. Of note, increased autophagy appears to be essential for NETosis and is reduced by HCQ [85].

Deficiency in either DNAase I or C1q, both necessary for NETs degradation and clearance, promotes persistent autoimmune stimulation by nuclear autoantigens [86].

Determination of baseline neutrophil to lymphocyte ratio, of circulating NETs remnants and of ANETA have been proposed as biomarkers to predict activity and outcomes in LN [87].

##### Monocyte/Macrophage

Monocytes and macrophages are important players in the pathogenesis of LN.

Infiltration of kidneys with these cells is associated with more severe disease and an increased risk of evolution towards ESRD [88].

Bulk transcriptome data identified differentially expressed genes and activation of IFN-I signaling in correlation with the abundance of infiltrating macrophages, both in glomeruli and in tubule-interstitium [89]. Macrophages involved in LN are characterized by a phenotypic change, from inflammatory patrolling monocytes to phagocytic and then to APCs capable of secreting complement components [90]. This evolution results in dysfunctional phagocytosis and an impaired removal of apoptotic cells, aggravating renal damage [91].

High expression of the Sphingosine-1-phosphate (S1P) receptor (S1PR1) and activation of the S1P/S1PR1 axis promotes macrophage accumulation and polarization towards the M1 pro-inflammatory phenotype through NLRP3 inflammasome [92].

Tissue Moesin deficiency has been associated with lupus-like nephritis, with an accumulation of chemokine (C-X-C motif) ligand (CXCL)13-producing patrolling monocytes and macrophages due to reduced migration to S1P [93]. Lymphangiogenesis, which appears to occur in LN but not in normal kidneys, may facilitate the infiltration of LN-specific monocytes [94].

Consistent with the important role played by monocytes, urinary levels of Monocyte chemoattractant protein 1 (MCP-1), which promotes monocyte migration to the kidney, are higher in individuals with active LN and correlate with early activity of LN, facilitating identification of “silent” LN [95].

##### Lymphocyte

A selective accumulation of T and B cells occurs in LN, with peculiar features. T follicular helper (TFH) and Th cells are an important pathogenic subset of CD4^+^ T cells in SLE. An increased TFH/Treg ratio can be observed in the peripheral blood of patients with active LN, especially in classes III and IV, and immunohistochemistry has revealed TFH1 cell infiltration in kidneys with LN [96]. A specific subset of infiltrating C-C chemokine receptor (CCR)4^+^ TFH lymphocytes seems to be involved in LN pathogenesis and T cell receptor repertoire appears to be relatively restricted, suggesting an oligoclonal expression of intrarenal lymphocytes [97]. Pathogenic TFH cell accumulation and function have been recently shown to depend on programmed death ligand (PD-L)1 and IL-4 in basophils, which can induce a transcriptional program leading to TFH2 cell differentiation [98].

The SLAM-associated protein (SAP) regulates TFH and Th function by binding to the co-stimulatory signaling lymphocyte activation molecule family (SLAMF) receptors that mediate interactions between T and B cells. SAP and SLAMF play a key role in Th-dependent B cell maturation into autoantibody-producing plasma cells (PC) in SLE. SAP expression is increased in LN in infiltrating T cells, including the TFH-like CD4^+^ and effector CD8^+^ T cells Furthermore the frequency of SAP^+^Th in circulation appears to correlate with disease activity and the presence of LN [99].

Another important T cell subset is represented by double-negative T cells, which are expanded in active SLE [100] and associated with high IL-17 levels, supporting production of autoantibodies [101].

On the other hand, Sm-specific Tregs (Sm-Tregs) have been shown to suppress disease. An HLA-DR15 restricted immunodominant Sm-T cell epitope suppresses Sm-specific pro-inflammatory responses in vitro and disease progression in a humanized mouse model of LN [102].

Progress in characterizing B lymphocytes has also been made. Memory B cells show a mitochondrial dysfunction in SLE. Reduced expression of an oxidative phosphorylation-regulating gene, Peroxiredoxin 6, has been found in SLE B cells and causes an upregulated mitochondrial respiration and increased antibody production [103]. Interestingly, the oxidative phosphorylation inhibitor IM156 inhibits activated B cells by regulating mitochondrial membrane potential and also blunts LN in the NZB/W F1 mice [104].

Abnormalities in lipid metabolism and mammalian target of rapamycin (mTOR) signaling also seem to play a role in the deranged immunometabolism observed in SLE [105].

Tertiary lymphoid structures (TLS), clusters of immune cells which organize in nonlymphoid tissue within renal interstitium, play an important role in the pathogenesis of LN and correlate with tubulointerstitial inflammation, higher immunological activity and clinical severity. This process is triggered by the local production of chemokines such as CXCL13 and results in an increased local production of IFN-I and also anti-dsDNA, anti-Sm, and anti-RNP. Of interest, the resolution of TLS occurs after inhibiting B-cell activating factor (BAFF) in animal models [106]. The role of tubular epithelial cells (TEC) in stimulating the formation of TLS is described in Section 3.4.1.

##### Dendritic Cell (DC)

Plasmacytoid DCs (pDCs) are the master producers of IFN-I in response to ICs and play a key role in TLR-mediated development of renal inflammation, autoimmunity, and fibrosis [107].

A novel population of inflammatory DC (infDC) appears to be differentially expressed in the LN kidney. These cells strongly express Fc receptor γ-chain, especially infiltrate periglomerular regions, and are adjacent to intrarenal CD3^+^ T cells, suggesting an interaction with these cells [108].

A strong interaction with CD4^+^ T cells is also typical of another recently characterized DC subset, CD163^+^DC (DC3s), which is enriched in LN and correlates with severity. DC3s contribute to intrarenal T cell expansion, effector T cell activation and polarization towards the Th1/Th17 phenotype. Of interest, injured proximal TECs may play a role in triggering DC3 recruitment within LN kidneys [109].

The autophagy-lysosome pathway has been identified as a potential mechanism involved in DC dysregulated maturation which is typical of SLE. TLR 9 seems to be involved in the activation of autophagy and lysosome acidification through the TRAF6-cGAS-STING pathway, making this a potential therapeutic target to modulate DC function [110].

Mitophagy dysfunction is another critical aspect in the development of LN and a novel mitophagy inducer, UMI-77, mitigated histological damage in the murine model of LN by inhibiting DC proinflammatory phenotypes. This drug also restored mitochondrial function in myeloid cells from patients with LN in vitro [111].

A recent study has demonstrated that IgA autoantibodies against a major SLE autoantigen, Sm ribonucleoproteins, play a role in IC-mediated activation of pDCs, which express the IgA-specific Fc receptor, FcαR. IgA1 autoantibodies appear to synergize with IgG in RNA-containing ICs in eliciting a robust IFNα response by pDCs [112].

Finally, recent evidence suggests that autotaxin, an enzyme that catalyzes the production of lysophosphatidic acid in the extracellular space, is produced by pDCs and correlates with levels of IFN-I. Autotaxin is increased in serum and urine of patients with LN and may represent an important biomarker [113].

### 3.4. Role of Kidney Resident Cells

Autoimmunity alone cannot lead to kidney damage without the essential contribution of resident cells, which are prone to significant functional changes due to chronic inflammation [114]. Several studies have expanded evidence of the important role of kidney resident cells in the pathogenesis of LN. The TECs, glomerular endothelial cell (GEC), MCs, podocyte, and parietal epithelial cell are exposed to ICs and inflammatory cytokines and each cell type actively contributes to inflammatory milieu in LN [115].

Endoplasmic reticulum stress has recently emerged as a shared mechanism of damage of renal resident cells in LN. For example, it can induce podocyte apoptosis, promote the secretion of inflammatory mediators by MCs, expression of adhesion molecules in GECs and apoptosis of TECs [116].

We will briefly analyze the most recent evidence on the role of each cell type.

#### 3.4.1. Tubular Epithelial Cells (TEC)

**Figure 3:**
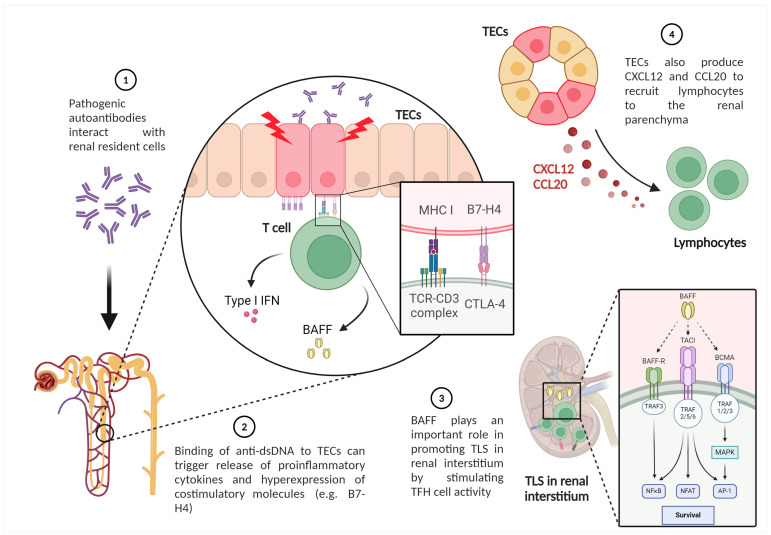
Activated or damaged renal tubular epithelial cells exert important immunological effects in LN (created with BioRender.com). Legend. TECs—tubular epithelial cells; BAFF—B cell activating factor; MHC—major histocompatibility complex; TLS—Tertiary lymphoid structures; TCR—T cell receptor; IFN—interferon.

Tubulointerstitial lesions have been recognized as an important component in the pathology of LN. TECs modulate interstitial milieu, promoting T cell infiltration and TLS formation. Loss of TEC integrity has been associated with intrarenal activation of adaptive immunity through several mechanisms, as illustrated in Figure 3. The binding of anti-dsDNA to TECs can directly trigger the release of proinflammatory cytokines. Furthermore, TECs express the costimulatory molecule B7-H4, which can activate T cells and secrete IFN-I and BAFF [117]. Even an autocrine loop of BAFF with its receptors on TEC has been demonstrated. This mediator plays a crucial role in promoting TLS in LN by stimulating TFH activity [115]. In addition, TECs also produce CXCL-12 and C-C motif ligand (CCL)20 to recruit lymphocytes to the renal parenchyma, along with several pro-inflammatory and pro-fibrotic factors [118].

#### 3.4.2. Podocyte

As observed for TEC, also podocytes can be directly damaged by autoantibodies in SLE and are involved in the immunological process. Injured podocytes activate innate immunity through the expression of TLRs and also trigger T cells through upregulated MHC and costimulatory molecules (CD80/CD86), acting as APC. Podocytes also contribute to crescent formation, together with parietal epithelial cells [119,120].

#### 3.4.3. Mesangial Cell (MC)

MCs respond early to IC deposition in LN. Anti-dsDNA antibodies, serum, or plasma from patients with LN have been shown to activate multiple signaling pathways such as JAK/STAT/SOCS, PI3K/AKT, and MAPK, inducing proliferation, expression of proinflammatory cytokines and profibrotic factors [121].

Also infiltrating inflammatory cells can stimulate MCs hyperproliferation and production of extracellular matrix, which contribute to glomerular fibrosis. Macrophages modulate the proliferation of MCs by interacting through the CXCL 12/dipeptidyl peptidase 4 axis. Of interest, treatment with linagliptin inhibited MC proliferation and reduced urinary protein levels in LN mice [122].

Renal deposition of Pentraxin 3 correlates with proteinuria and inflammation in LN and facilitates MC proliferation through the MAPK/ERK1/2 signaling pathway. Protopanaxadiol can effectively inhibit the abnormal proliferation of MCs and improve proteinuria by inhibiting this pathway [123].

MCs also exert phagocytosis, APC function and proinflammatory effects, aberrantly participating to a renal-resident immune response which triggers a second wave of mesangial damage after the initial phase of IC deposition [124].

#### 3.4.4. Glomerular Endothelial Cell (GEC)

Deposition of ICs within the subendothelial area appears to affect GEC functions, including activation of apoptosis, inhibition of angiogenesis [125], and dysregulation of the coagulation/fibrinolysis system [126]. Increased expression of TLR3 on GEC mediates the production of IFN-I and consequently of IFN-stimulated genes. Among these, IFN-stimulated exonuclease gene (*ISG 20*), coding for an antiviral effector protein, was intensely expressed in biopsy specimens from proliferative LN and regulated CX3CL1 production, promoting glomerular inflammation [127].

Renal endothelial-podocyte crosstalk, an important aspect of LN, is mediated by activation of the mTOR signaling pathway. Glomerular activation of the mTOR pathway was significantly increased in patients with endocapillary hypercellularity and podocyte damage [128].

## 4. The Role of Renal Biopsy and of New Biomarkers in the Management of LN

Despite progress in the discovery of diagnostic and prognostic biomarkers, renal biopsy remains essential in clinical practice to diagnose LN, monitor its activity, and provide prognostic elements [129].

### 4.1. Renal Biopsy

#### 4.1.1. Renal Biopsy as a Diagnostic Tool

Renal biopsy remains an essential diagnostic tool to classify renal lesions and provide information about activity or chronicity, all crucial aspects to guide therapy. A revised renal classification of the International Society of Nephrology/Renal Pathology Society classification, published in 2018, eliminated some class subdivisions of previous versions (class IV segmental and global) and proposed the modified National Institutes of Health chronicity index to define the degree of activity in all classes [130]. A large study validated this new classification in a Chinese population in 2020, showing that fibrous crescents, tubular atrophy/interstitial fibrosis and this chronicity index reliably predicted a composite renal outcome [131].

Despite such improvements, however, four important histological patterns are not included in this classification: glomerular crescents, lupus podocytopathy, tubulointerstitial lesions, and thrombotic microangiopathy (TMA). These peculiar lesions and their underlying pathogenesis deserve further study and may require specific therapies as compared to “traditional” classes [132]. The presence of TMA, for example, can often be resistant to first-line IS therapy and might benefit from plasma exchange or anti-complement agents [133]. A wider classification of LN has been proposed on this basis [132].

Another aspect is the high burden of premature arteriosclerotic renal lesions which has been demonstrated in a recent study, suggesting that these develop two decades earlier in LN patients compared to their healthy peers and are overlooked by pathologists in half of cases [134].

A recent study has underlined that the assessment of interstitial inflammation in the entire cortical parenchyma (and not only in unscarred areas) allows for the identification of patients at risk for CKD progression in LN, in contrast to the current classification of interstitial inflammation [135].

Finally, inter-rater reliability remains a limit of current classification. The inclusion of molecular classifiers into histologic analysis has been proposed to improve diagnostic precision and identify more precisely kidney biopsy phenotypes and therapeutic targets. For example, an abundance of transcripts related to glomerular fibronectin, secreted phosphoprotein-1, and galectin-3 appears to correlate with disease activity and response to treatment [136]. Identifying different genetic landscapes of LN based on patterns defined by the expression of sets of “hub genes” is an evolving perspective, which will likely change the role of traditional analysis of renal biopsy, as discussed in Section 8 [137,138,139].

#### 4.1.2. Renal Biopsy as a Prognostic Tool

Currently, only serum creatinine and proteinuria can predict long-term evolution in LN. For example, achieving a reduction of proteinuria below 0.5 g/d with preserved renal function—complete remission (CR)—has prognostic value and a cut-off level of 0.7 g/d at month 12 has been associated with stable renal function after 7 years [140]. However, this parameter has limited specificity: proteinuria can express either residual active inflammation or onset/evolution of chronic lesions and histological activity may persist despite reduction of proteinuria [141]. This makes interpretation difficult especially in cases of partial renal response (PRR), expressed by a reduction of 50% in proteinuria to sub-nephrotic levels.

Potential significant dissociation between clinical and histological findings makes renal biopsy still pivotal in managing LN, both for initial diagnosis and as a tool for response assessment (repeat or per-protocol biopsies). The prognostic value of tubulo-interstitial inflammation on 10-year risk of ESRD and of crescents on mortality has been recently confirmed by a real-world Chinese study [142] and similar results have been reported in other ethnicities [143]. Ideal timing for per-protocol repeat renal biopsy is debated [144]. An early approach (6 months after diagnosis) has been proposed to verify the response to induction, as earlier detection of poor prognostic signs in patients without clinical deterioration might improve outcomes [145]; alternatively, a renal biopsy could be performed after 1–2 years to assess treatment efficacy and modulate the duration of maintenance therapy [146].

The “ReBIOLUP” randomized study is currently evaluating the role of per-protocol repeat biopsies in guiding therapy, especially at the initial phase (incident LN), to prevent residual inflammation and long-term nephron loss [147]. Renal biopsy remains essential for the concept of “treat to target” in LN, as CR can only be reliably defined through histology.

Findings at the second per-cause biopsy, but not at the first one, provided histological predictors of long-term risk of ESRD in persistently active or relapsing LN in a large Italian study [148,149].

### 4.2. Potential Biomarkers

There is an unmet need for non-invasive biomarkers of disease activity to inform treatment responses and guide therapy.

Several biomarkers hold promise towards optimization of LN management, with the use of integrated omics and sets of biomarkers which could, in the future, limit the need for renal biopsies [150]. The main ones available are summarized in Table 2.

Urinary biomarkers are especially interesting as they may better reflect the compartmentalized renal response in LN, unlike serum studies, which are generally less kidney-specific [4]. Increasingly widespread use of cutting-edge omic technologies is leading to the discovery of many novel potential biomarkers for LN [151].

**Table 2:** Main serum and urinary candidate biomarkers for LN.

Biomarker	Biological Fluid	Associations	References
u-Gal-3BP	Urine	Histological disease activity	[152,153,154]
MCP-1	Urine	Clinical severity	[155,156]
TWEAK	Urine	Diagnosis	[157]
CAM	Urine	Clinical severity	[158,159,160]
miR146a miR135b	Urine	Histological disease activity; Response to therapy	[161,162,163]
Axl	Serum	Diagnosis; Clinical severity; Response to therapy; Prognosis	[164,165,166]
HE4	Serum	Diagnosis	[167,168]
IGFBP-2	Serum	Diagnosis; Clinical severity; Organ damage	[169]
miR	Serum	Diagnosis	[170,171]
IL-17 and IL-18	Serum	Clinical severity and histological activity	[172,173,174]
sTNFRII	Serum	Clinical severity and histological activity Organ damage	[175,176]
Adipokines	Serum	Clinical severity; Atherosclerotic organ damage and insulin-resistance	[177,178,179,180]
BAFF and APRIL	Serum	Clinical severity and histological activity; Response to therapy	[181,182,183,184,185,186,187]
Syndecan-1	Serum	Clinical severity and histological (tubulointerstitial) activity	[188]

Legend. u-Gal-3BP—Urinary galectin 3 Binding protein; MCP-1—Monocyte chemoattractant protein 1; TWEAK—TNF-like weak inducer of apoptosis; CAM—cell adhesion molecule; MiR—MicroRNA; HE4—Human epididymis protein 4; IGFBP2—Insulin-like growth factor-binding protein 2; BAFF—B cell activating factor; APRIL—a proliferation inducing ligand.

#### 4.2.1. Urinary Biomarkers

##### Urinary Galectin 3 Binding Protein

This β-galactosidase-binding lectin involved in apoptosis, inflammation, and fibrosis, is often released into biological fluids, including urine, from the surface of injured and inflammatory cells. It appears to be a diagnostic or prognostic biomarker for several autoimmune disorders and kidney disease at their early stages [152]. In the setting of SLE, it can discriminate against patients with active LN from active non-renal and inactive patients. Furthermore, it correlates with histological activity, with higher levels detected in proliferative (class III/IV) and membranous LN than in mesangial (class II) form [153]. This profile makes it a potential surrogate biomarker of renal biopsy [154].

##### Monocyte Chemoattractant Protein-1 (MCP-1)

Also referred to as CCL2, it is a key mediator of innate immunity involved in kidney disease-related inflammation and its expression directly correlates with the severity of nephropathy. MCP-1 can induce migration and infiltration of lymphocytes, NK cells, and monocytes [155]. Of interest, CCR2, the receptor of MCP-1, is highly expressed on the surface of peripheral γδT cells, which accumulate in renal tissue in LN [156].

##### TNF-like Weak Inducer of Apoptosis (TWEAK)

TWEAK is an important member of the TNF superfamily. Upon binding to its receptor, fibroblast growth factor-inducible 14 (Fn14), it regulates inflammatory and fibrotic processes. Dysregulation of the TWEAK/Fn14 axis probably plays a significant role in LN [157].

##### Cell Adhesion Molecules (CAM)

Several urinary CAMs released from cell membranes could serve as biomarkers, such as vascular CAM1 (VCAM-1), activated leukocyte CAM (ALCAM, or CD 166), kidney injury molecule 1 (KIM1), neutrophil gelatinase-associated lipocalin (NGAL) and soluble CD163 receptor (sCD163) shed by M2 macrophages. Overall elevated urinary levels of these CAMs can reliably distinguish renal from non-renal SLE and active from inactive LN.

ALCAM binds to the CD6 receptor on T lymphocytes, resulting in activation and recruitment of these cells into renal tissue. Levels above threshold value of 270 ng/mg can identify active LN and are positively correlated with SLEDAI and negatively with complement C3 and C4 fractions [158]. An ELISA assay for urinary ALCAM may represent a convenient tool for early detection of LN and of renal flares, even facilitating home-monitoring of LN activity [159].

NGAL has shown the best performance in predicting clinical response 6 month after induction therapy among these CAMs. Furthermore, NGAL urinary levels are especially high in class IV LN; in this setting they positively correlate with anti-ds-DNA and proteinuria and negatively correlate with serum albumin and C3 fraction level [160].

##### MicroRNA (miRs)

A recent meta-analysis identified 4 urinary miRs isolated in extracellular vesicles (EV) related to several pathways involved in LN [161]. Among these, miR146a baseline levels appear to be associated with albuminuria and renal flares [162], whereas miR 135b would identify responders to IS therapy [163].

#### 4.2.2. Serum Biomarkers

##### Axl

Axl is an important tyrosine kinase receptor found in myeloid cells, with a role in immune innate system regulation and in the clearance of apoptotic cells [164]. Elevated sAxl levels are found in LN and correlate with renal activity. Furthermore, worse chronicity scores are associated with elevated post-treatment sAxl levels. These features make Axl a potential biomarker to monitor renal response to IS therapy and the progression of renal damage in LN [165]. Persistently high sAxl levels after treatment completion may suggest the need for intensified treatment [166].

##### Human Epididymis Protein 4 (HE4)

HE4 is commonly used as a tumor marker, especially for ovarian cancer, and elevated serum levels can also be found in CKD [167]. Increased serum HE4 levels have been recently found to be independently linked to a higher risk of developing LN. However, renal dysfunction might reduce HE4 clearance, complicating the interpretation of elevated serum HE4 levels in this setting [168].

##### Insulin-like Growth Factor-Binding Protein 2 (IGFBP2)

IGFBP-2 is a member of the IGFBPs family, with a potential role as a biomarker for several malignant tumors [189]. Patients with LN are characterized by elevated levels of serum IGFBP-2 compared to CKD patients and healthy controls, making it a potential diagnostic biomarker reflecting renal and global immunological activity. Serum IGFBP-2 can also correlate with serological (anti-dsDNA antibody titers and complement levels) and renal parameters (serum creatinine and urine protein-to-creatinine ratio), in addition to the renal chronicity index [169].

##### miR-21

Inhibition of miR-21 in T and B cells may improve multiple organ damage in SLE, suggesting a pathogenetic role [170]. A more intense expression of miR-21 in active LN than in LN-absent and inactive LN patients makes it an interesting tool. However, there was no significant correlation between miR expression and LN pathological classes [171].

##### IL-17 and IL 18

Some cytokines are especially associated with LN and could represent biomarkers. Circulating IL-17 levels are higher in patients with active LN than in patients with inactive LN or controls. IL-17-producing cells are present in glomeruli from LN patients and are associated with complement activation and increased immunoglobulin deposition. IS therapy appears to reduce IL-17 levels in active LN and a significant correlation exists between LN exacerbations, elevated serum levels of IL-17 and IL-23, and SLEDAI [172]. Another important cytokine is IL-18, which plays a major pathogenetic role in LN by promoting cytokine imbalance towards Th1-type immune response [173]. Interestingly, heightened levels of IL-18 and its binding protein (IL-18 BP) have been found in both serum and glomeruli of patients with active LN [174].

##### Soluble TNF Receptor 2 (sTNFR2)

Soluble TNF receptor 2 (sTNFR2) is mainly expressed on Treg cells and plays an important role in regulating apoptosis and proliferation of thymocytes and cytotoxic T-cells. sTNFR2 levels are higher in SLE patients than in HC and they correlate with disease activity and evolution, decreasing after IS treatment [165]. In another recent study sTNFR2 levels correlated with chronic index scores in renal biopsies and long-term eGFR deterioration [175]. Overall sTNFR2 may represent a biomarker of therapy response, LN activity, and prognosis [176].

##### Adipokines

LN patients are characterized by endothelial dysfunction and premature atherosclerosis and proinflammatory adipokines are involved in this process and may represent biomarkers.

Serum but not urine resistin has been correlated with SLE disease activity, insulin resistance [177], and renal dysfunction in LN [178].

Adiponectin, leptin, and visfatin levels, along with Homeostasis Model Assessment-Insulin Resistance (HOMA-IR) index, were higher whereas brachial artery flow-mediated vasodilatation was lower in LN cases than in SLE without renal involvement in a recent study [179].

Higher levels of adiponectin and leptin were confirmed to be higher in SLE patients and positively correlated with SLEDAI and LN, as well as with greater BMI and CRP levels [180].

##### B Lymphocyte Activating Factor (BAFF) and “a Proliferation-Inducing Ligand” (APRIL)

BAFF is a B cell survival factor which supports autoreactive B cells and is strongly involved in SLE pathogenesis. In addition to autoimmunity, it is also involved in adipogenesis, atherosclerosis, and neuroinflammation [181].

BAFF is overexpressed in SLE, a significant correlation between serum BAFF levels and disease activity has been demonstrated [182] and renal tissue expression of BAFF and its receptors is associated with class IV proliferative LN [183].

Serum BAFF and IFN-I have been proposed as biomarkers to stratify patients; high BAFF identifies those with LN and high IFN-I marks those with blood and skin manifestations. Levels of these two pivotal cytokines would guide therapy with appropriate biologics [184].

The TNF superfamily member “a proliferation-inducing ligand” (APRIL) plays a late role in humoral immunity at the level of antibody-producing PCs and is considered a target to dampen autoantibody production [185]. High serum levels of APRIL characterize severe proliferative LN with specific lesions such as endocapillary proliferation, neutrophil infiltration, and fibrinoid necrosis.

Interestingly, both BAFF and APRIL levels are associated with response to IS therapy, although in a different way. Low baseline BAFF levels (<1.5 ng/mL) seem to predict treatment response in LN, especially in proliferative forms, whereas high APRIL levels (>4 ng/mL) strongly predict treatment failure. However, only APRIL levels decrease after induction treatment in responders and its intrarenal mRNA levels would be associated with resistance to treatment, proteinuria, and histological activity [186,187]. These pivotal mediators may help predict response to IS therapy and identification of resistant cases.

##### Syndecan-1 and Other Glycocalyx Components

Glycocalyx is a gel-like layer at the interface between endothelial cells and the bloodstream, composed of proteoglycans and glycosaminoglycans, glycoproteins, and plasma proteins on the luminal side [190]. This structure can be damaged by multiple noxae including inflammation, which determines the shedding of glycocalyx components. As shedding is an early process associated with endothelial activation and damage, some of these circulating molecules act as “danger-associated molecular patterns” and have been proposed as biomarkers of cardiovascular disease and renal damage in LN. Sydecan-1 is probably the most promising of these molecules, as increased levels are observed in active LN compared to remission and they correlate with anti-dsDNA titer, SLEDAI-2 K, proteinuria, serum creatinine, and severity of interstitial inflammation [188].

Circulating levels of hyaluronan and thrombomodulin have also been associated with LN and represent potential biomarkers [191].

## 5. Immunosuppressive (IS) Therapies in LN

**Figure 4:**
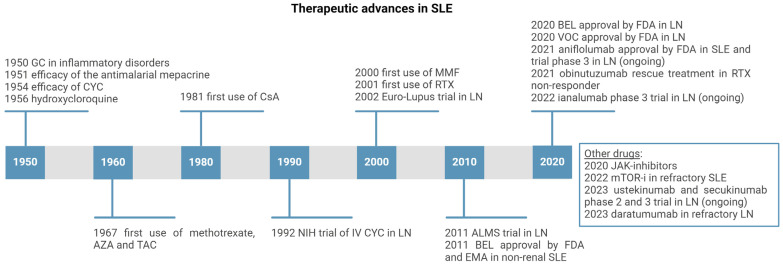
Timeline of the development of main immunosuppressants employed in treatment of SLE and LN (created with BioRender.com). Legend. GC—glucocorticoid; CYC—cyclophosphamide; CsA—Cyclosporin A; AZA—Azathioprine; TAC—Tacrolimus; MMF—mycophenolate mofetil; RTX—Rituximab; BEL—Belimumab; VOC—voclosporin; JAK—Janus Kinase; mTOR-i—mammalian target of rapamycin inhibitor.

The therapeutic landscape of LN has been rapidly changing over the last few years (Figure 4). We will first analyze conventional IS therapies, which still represent the backbone of IS therapy, along with RTX, and then focus on new FDA-approved drugs, BEL and VOC, and other drugs under investigation in phase II-III trials.

### 5.1. Conventional Is Therapies

The standard of care of LN has been based on high-dose GCs combined with either cyclophosphamide (CYC) or mycophenolate mofetil (MMF) for induction treatment and on low-dose GC coupled with either MMF or azathioprine (AZA) for maintenance therapy.

We will review the most recent studies on conventional IS therapies for the induction and maintenance phase. Rituximab (RTX), which has been mainly used as rescue therapy for resistant or relapsing forms of LN, will be separately analyzed.

#### 5.1.1. Induction Therapy

A systematic meta-analysis was recently performed on the control arms of 50 RCTs, in which LN was treated with GCs in combination with mycophenolic acid (MPA) analogs or CYC. A dose-response gradient was observed between induction steroid dose and all outcomes at six months. A higher steroid dose was associated with better renal outcomes including CR, but at the price of increased infections and mortality [192]. In a post-hoc analysis of a retrospective multicenter Egyptian study, an increase in mortality was shown with every gram increase in cumulative methylprednisolone (MP), especially in doses exceeding 2.75–3.25 g [193]. Interestingly, the addition of low-dose steroid pulses (MP 125 mg) to each fortnightly dose of 500 mg of CYC within Eurolupus Nephritis Trial (ELNT) protocol appears to enhance response and reduce the need for oral GC in LN of class III, IV and V [194]. This evidence reinforces the concept of using steroid pulses at the lowest possible dose as induction [195].

CYC still remains one of the cornerstones of traditional induction therapy and it is usually administered according to ELNT protocol [196]. Long-term effectiveness in the preservation of renal function [197] and an acceptable profile of side effects [198] are its strengths. Furthermore, it is still the preferred agent in special scenarios of SLE with life-threatening extrarenal manifestation [199] (e.g., central nervous system or pulmonary involvement) and in rapidly progressive LN [200].

Even though MMF was shown to be effective as an alternative induction therapy to CYC, actual superiority of MMF in African Americans and Hispanics has been recently confirmed [201]. Furthermore, a meta-analysis of 18 trials on a Chinese population also showed that MMF was significantly more effective than CYC induction in proliferative LN, with a more favorable profile of side effects [202]. Interestingly, differential effects of CYC and MMF on B cell subsets may partly account for improved response to MMF in high-risk ethnicities. MMF appears to be associated with an earlier reduction of circulating plasmablasts and PCs compared with CYC, whereas CYC would determine preferential depletion of less mature B cells (naïve and pre-switched memory B cells) compared with MMF [203]. An important common limit of CYC and MMF is that neither drug had significant effects on class-switched memory B cells, which are typically resting during active LN but can reactivate and trigger disease relapse in the maintenance phase, even in patients who responded to induction [204].

Another class of drugs which can be employed as induction therapy is represented by calcineurin-inhibitors (CNI). Both tacrolimus (TAC) and cyclosporine A (CsA) have long been used in LN but TAC has been the preferred agent over the last few years [205].

Several RCTs have demonstrated non-inferiority of TAC to MMF or CYC for induction therapy of LN and a low-dose combination of TAC and MMF (“multi-target therapy”) has even been shown to outperform CYC pulses in inducing LN remission in Chinese patients. Furthermore, TAC can be an alternative option for SLE patients who are intolerant or refractory to conventional IS therapy due to its different safety profile and mechanism of action [206]. The availability of VOC is now allowing another type of multitarget therapy (VOC + MMF) (reviewed in Section 5.2.2).

A recent meta-analysis (6 RCTs, including 1.437 patients) showed that different types of multitarget therapies such as VOC + MMF and TAC + MMF achieved a higher CR rate than monotherapy with MMF or CYC, but at the price of numerically higher cases of infections including pneumonia [207].

The concept of multitarget therapy with VOC and also BEL will be further discussed in Section 5.2.

#### 5.1.2. Maintenance Therapy

Accumulating evidence has demonstrated MMF maintenance is associated with a lower risk of disease relapse compared with AZA, making it the drug of choice in this setting [208].

Two important studies published in the last few years have focused on the duration of maintenance therapy with MMF.

A recent RCT, “Weaning of immunosuppressive therapy in LN” (WIN study), compared a protocol of withdrawal of IS therapy after 2–3 years of maintenance therapy versus a standard one of longer duration in proliferative LN, demonstrating that the former was associated with more frequent severe flares at 24 months [209].

In a recent multicenter, open-label, randomized trial conducted in the USA, maintenance with MMF was withdrawn in stable patients (SLEDAI < 4) after 2 years. The increase in clinical disease reactivation by 60 weeks of randomization with MMF withdrawal was 7%, whereas infections were more frequent in the MMF maintenance group. The Authors concluded that MMF withdrawal was not significantly inferior to drug maintenance [210].

Overall optimal duration of maintenance therapy remains uncertain, although the availability of stronger induction protocols (i.e., “triple therapy” with either BEL or VOC) appears to allow a much more rapid steroid tapering and potentially also a global maintenance therapy of reduced intensity [192].

#### 5.1.3. The Role of Rituximab (RTX)

Even if formal supporting evidence is lacking, RTX continues to be used off-label in LN for resistant or relapsing forms and as a steroid-sparing agent, based on a considerable amount of anecdotal clinical data, with a success rate of around 50–70% in proliferative classes [211]. However, current guidelines do not indicate it as a first-line induction drug [212], mainly as a result of a landmark LUNAR study, which did not prove its efficacy as an induction add-on therapy on top of MMF and GC [213]. A likely cause of inadequate response to RTX in this study was incomplete B cell depletion, which might correlate with an inability to reduce tubulointerstitial lymphoid aggregates [214]. A recent real-world Japanese study has underlined that RTX efficacy is heavily patient-driven [215] and that it can trigger production of antibodies which blunt its action [216].

Despite these limits, RTX remains an important biological agent in the management of LN and also its role as an induction agent is still a matter of debate. A recent Bayesian network meta-analysis on Chinese populations (1566 patients from 19 studies) comparing RTX, TAC MMF, and CYC as induction therapies concluded that RTX and TAC were the most effective drugs in order to achieve CR in LN, with RTX determining the highest risk of infection and TAC the lowest one [217].

Obinutuzumab, a new humanized and more potent anti-CD20 monoclonal antibody, may be effective in RTX non-responders and will be discussed in Section 5.3.2.

### 5.2. New FDA-Approved Drugs

Conventional regimens described in the previous section are generally effective and with an acceptable profile of toxicity. However, a significant proportion of patients experience renal or extra-renal flares (30–40%) and rates of long-term progression towards ESRD have remained unchanged over the last decades (10–30%). This has prompted a search for new treatments, which have exponentially grown especially over the last few years. The main recent phase III trials concerning LN therapy are outlined in Table 3.

Since 2020 two drugs were approved by the FDA for induction, BEL and VOC, which can each be added to MMF/MPA and GC, leading to two new IS schemes of “triple therapy”. This multi-target approach adds to traditional “dual therapy” protocols (CYC and steroid; MMF/MPA and steroids) and is paving the way for a more individualized, tailored induction and also for a new vision of induction [224]. Starting with a double therapy and subsequently switching to a triple one if the response is inadequate (“step-up approach”) or, on the contrary, starting with triple therapy and then decreasing it to a double one (“step-down approach”) represent interesting new algorithms requiring further investigations [224,225]. This evolution is leading to a change from the traditional sequential “induction-maintenance” scheme to the concept of continuous, combined triple therapy in selected patients [226]. We will analyze BEL and VOC, which have made this progress possible.

**Figure 5:**
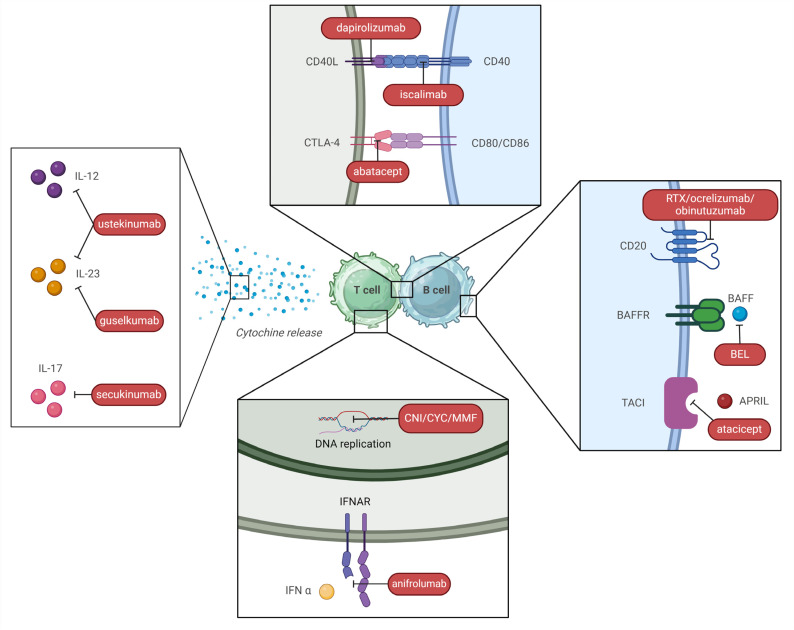
Different mechanisms of action of immunosuppressants used in SLE treatment (created with BioRender.com). Legend. CNI—calcineurin inhibitor; CYC—cyclophosphamide; MMF—mofetil mycophenolate; BAFF—B cell activating factor; APRIL—a proliferation-inducing ligand.

An overview of different mechanisms of action of these drugs and other immunosuppressants is outlined in Figure 5.

#### 5.2.1. Belimumab (BEL)

BEL is an anti-BAFF monoclonal antibody (Figure 5). After an initial FDA approval for active non-renal SLE in 2011, the drug was approved as an induction treatment for active LN in 2020, in addition to either MMF or CYP (ELNT) with steroid, based on the results of the BLISS-LN study [218]. This RCT evaluated 448 patients with class III, IV, and V LN at 2 years. Primary efficacy renal response was defined as a GFR of at least 60 mL/min or a GFR level above 20% of baseline level and a urinary protein-to-creatinine ratio < 700 mg/mg. Of note, steroid therapy had to be reduced to prednisone (PDN) 10 mg or less by week 24. This primary endpoint was met in 43% of patients with BEL arms vs. 32% of the placebo. Furthermore, a lower risk of renal events or death was observed in treated patients [227]. Overall current evidence suggests BEL can be preferred within an induction triple therapy in severe forms of LN with important renal function impairment (lack of nephrotoxicity) or in patients with a history of renal (or extra-renal) flares, in which prevention of further flares is essential to preserve renal function; on the contrary, BEL is probably less effective in LN with important proteinuria (>3 g/d), in which VOC seems to be a better alternative [224,228].

An open-label extension of the BLISS-LN study has actually demonstrated that upfront addition of BEL to MMF or CYP as induction therapy may improve renal outcomes as compared with BEL sequential introduction, reinforcing its role as therapy ab initio [229].

Other possible indications are the need to spare GCs [230] or the need to reduce the dose of MMF/MPA due to intolerance or side effects [231]. A recent Spanish study indicates that BEL reduces healthcare resource utilization, including hospital admissions, probably stabilizing disease activity [232].

BEL can also be employed within a sequential RTX-BEL scheme. In the BEAT LUPUS study, this approach significantly suppressed B-cell repopulation, reduced anti-dsDNA, and prevented flares caused by the post-RTX surge in BAFF levels in refractory SLE [219].

Finally, the “SynBioSe 2” multicenter phase III clinical trial (Synergetic B-cell Immunomodulation in SLE) is testing the effectiveness of association BEL + RTX combined with standard-of-care (SOC) as induction treatment, followed by BEL and steroid as maintenance treatment [233].

#### 5.2.2. Voclosporin (VOC)

The use of CNI, CsA, and TAC with MMF has long been proposed as multi-target therapy (Figure 5) in several studies performed mainly on Chinese populations [234]. VOC is a new generation CNI with several advantages compared to CsA and TAC: it is better absorbed and has a more consistent pharmacokinetic-pharmacodynamic relationship due to enhanced calcineurin binding and reduced metabolite load, making therapeutic drug monitoring unnecessary [235]. Furthermore, it does not determine metabolic side effects such as dyslipidemia and new-onset diabetes, and it is not nephrotoxic, although it can cause a transient decrease in GFR and arterial hypertension [236,237]. However, a direct head-to-head trial of VOC versus CsA or TAC has never been performed [238]. Phase III AURORA 1 trial (2021), which determined drug approval, was conducted in patients with class III, IV, or V LN and showed that the association of VOC and MMF met the primary endpoint of CRR in 41% of treated patients vs. 23% of the placebo arm. CRR included urine protein/creatinine ratio ≤ 500 mg/g and ≥GFR 60 mL/min and no rescue treatment. Among secondary endpoints, VOC determined a much faster reduction of proteinuria, which was lowered to ≤500 mg/d in less than 6 months. Of note, steroid tapering was remarkably rapid in the treatment arm, reaching PDN 2.5 mg/day by week 16 [220]. Phase III AURORA 2 extension trial showed stable renal function after 3 years of therapy [221] and no evidence of CNI nephrotoxicity at renal biopsy [236].

Due to this profile, VOC is revolutionizing protocols to treat LN, paving the way to new possibilities beyond background therapy with MMF and steroids. Apart from triple induction with either BEL or VOC on top of MMF/MPA and GC, as suggested by KDIGO guidelines [239], VOC-BEL sequential protocols and VOC and BEL combination therapy (four-drug induction) have been recently proposed but both deserve further study [240].

### 5.3. Drugs in Phase III Clinical Trials

#### 5.3.1. Anifrolumab

Anifrolumab, an anti-type 1 IFN receptor antibody (Figure 5), also showed promising results in the treatment of LN. After the “Treatment of Uncontrolled Lupus via the IFN Pathway” (TULIP) 2 trial, it was approved for SLE by the FDA in 2021 [241], whereas TULIP LN assessed its use as an induction therapy for LN (in addition to MMF and steroid) in 2022 [222]. Although the introduction of Anifrolumab did not result in a statistically significant superiority, numerical improvements as compared to placebo were reported for many secondary outcomes [242]. Of note, a post-hoc analysis showed that sustained GC tapering was possible in a significantly higher percentage of patients under anifrolumab (51% vs. 32%) with a PDN dose of 7.5 mg/d through week 52 [243]. The second-year extension of the TULIP LN trial confirmed superior renal response at week 104 with an intensified regimen [222]. A phase III trial is evaluating the association of Anifrolumab and MMF in class III or IV LN (ClinicalTrials.gov Identifier: NCT05138133).

#### 5.3.2. Obinutuzumab

This is a new humanized anti-CD20 monoclonal antibody [223,244] (Figure 5) which was initially employed as rescue therapy for RTX non-responders, with promising results in case series [245]. The NOBILITY clinical trial has shown that obinutuzumab improves renal outcomes when added to a MMF and GC, an effect maintained at 104 weeks. Profound and rapid B cell depletion appears to account for it [223]. A post-hoc analysis of the NOBILITY trial [244] has demonstrated superior preservation of kidney function and prevention of flares as compared to SOC in proliferative forms of LN. Furthermore, CRR was achieved in a higher proportion of patients at week 76 (38% vs. 16% *p* < 0.01), despite receiving a lower steroid dose (PDN ≤ 7.5 mg or less). Of note, this difference lost statistical significance at week 76, but the trend was maintained (*p* = 0.06).

#### 5.3.3. Ianalumab

Ianalumab is a human monoclonal antibody with a dual mechanism of action, based on binding to BAFF receptor and elimination of B cells expressing it with a mechanism of antibody-dependent cellular cytotoxicity. This monoclonal antibody was effective in reducing immunological activity in primary Sjogren syndrome [246]. A phase III trial including class III, IV, and V of LN is underway to assess Ianalumab and MMF as an induction therapy and will be completed by 2030 (ClinicalTrials.gov Identifier: NCT05126277).

#### 5.3.4. Sodium-Glucose Cotransporter-2 Inhibitors (SGLT2i)

This class of drugs is being increasingly employed in CKD and proteinuria, in which they represent an effective complement to renin-angiotensin inhibitors [247]. Furthermore, they are being investigated in several glomerulonephritis (GN) such as IgA nephropathy. Some evidence suggests that Sodium-glucose Cotransporter-2 inhibitors (SGLT2i) can alleviate podocyte damage in LN [248]. In a few, small trials they blunted proteinuria and had a moderate effect on blood pressure and BMI, without affecting immunological activity [249]. A recent USA multicenter cohort study investigated 1775 matched pairs of SGLT2i users and non-users within a SLE population and found that the former had a significantly lower risk of developing LN and of progressing to ESRD, in addition to reduced risk of heart failure and all-cause mortality [250]. SGLT2i are therefore starting to gain a role as nephro-cardioprotective drugs, in association with renin-angiotensin system inhibitors, even in the high-risk population of SLE patients, which were previously excluded from major trials due to concerns for risk of urinary infections [249].

### 5.4. Other Drugs

Several other drugs are being investigated in phase II and III trials and have provided promising results in LN, which deserve further confirmation.

#### 5.4.1. Ustekinumab and Secukinumab

IL 17/23 axis and CD3^+^ CD4^−^ CD8^−^ double negative Th17 cells have been increasingly recognized as key players in LN pathogenesis [251].

Ustekinumab, an IL 12 and IL 23 inhibitor, met the primary endpoint in a phase II trial [252] but results were not confirmed in a phase III trial [253].

Secukinumab, an anti-IL17 antibody, has been employed in cases of refractory LN [254] and is currently being assessed in phase III trials (ClinicalTrials.gov Identifier: NCT04181762) (Figure 5).

#### 5.4.2. Inhibitors of Mammalian Target of Rapamycin (mTOR)

Inhibitors of mammalian target of rapamycin (mTOR) complex activation plays a pivotal role in Treg cell dysfunction and decreased Treg/T helper ratio, a hallmark of SLE [255]. Two inhibitors of the mTOR signaling pathway, rapamycin and everolimus, have been employed as add-on treatments in small studies with promising results [256], especially in refractory SLE [257].

Sirolimus is being investigated in a phase III trial for LN (ClinicalTrials.gov Identifier: NCT04582136).

#### 5.4.3. Janus Kinases (JAK) Inhibitors

Janus Kinases (JAK) inhibitors have been investigated in SLE in recent years with mixed results. JAK and signal transducer and activation of transcription (STAT) pathways mediate downstream effects of receptors for multiple chemokines and cytokines including IFN-I, making JAK-STAT signaling blockade an attractive approach in SLE [258]. In a preclinical RCT on MRL/MpJ-Faslpr mice, Baricitinib, an oral selective inhibitor of JAK 1 and 2, did not affect proteinuria, GFR, and histological lesions but improved lymphadenopathy and serological activity [259].

Baricitinib was studied in two large multi-center phase-3 RCTs in 2023, in which it was employed in addition to a background therapy of MMF and GC. The drug met the primary endpoint (higher proportion of patients reaching an SLE Responder Index -4 response at week 52) but not key secondary ones, including steroid tapering, in SLE-BRAVE-I study [260]; furthermore, results were not replicated in SLE-BRAVE-II [261].

Tyrosine kinase 2 (TYK2), a member of the JAK kinase family of intracellular signaling molecules, is another target of interest [262]. Deucravacitinib, a small molecule which inhibits TYK2, yielded greater response rates than placebo in a phase II trial of SLE, including Lupus Low Disease Activity State and cutaneous and articular manifestations [263]. Ongoing phase III trials of baricitinib and deucravacitinib may provide more conclusive evidence in the upcoming years.

#### 5.4.4. Daratumumab

Daratumumab, an anti-CD38 monoclonal antibody, has been recently proposed as a monotherapy of refractory LN in a case study on six patients. In 5 out of 6 patients, the mean disease activity significantly decreased at 12 months after treatment, along with anti-dsDNA, IFNγ levels, proteinuria, and serum creatinine [264]. Other case reports have recently suggested the effectiveness of this drug in refractory LN [265] and anti-phospholipid syndrome (APS) [266].

## 6. Adjunctive Therapies beyond Immunosuppression

Management of LN also includes an adjuvant therapy which aims at treating frequent comorbidities of these patients, with a holistic approach. Cardiovascular disease, CKD, and infections currently remain the leading causes of mortality in SLE [267] and a recent systematic review and meta-analysis showed that patients with LN have a significantly higher risk of hypertension, dyslipidemia, and diabetes mellitus compared with those without it [268]. Therefore, control of key cardiovascular risk factors and introduction of nephro-cardioprotective drugs, such as ACE inhibitors or sartans, is recommended by all guidelines, including the most recent ones [205]. Management of drug-related side effects, such as GC-induced osteoporosis or obesity is equally important. We will briefly analyze the main therapies in this setting.

### 6.1. Control of Blood Pressure

This is a crucial aspect, which has an impact on both cardiovascular mortality and progression of CKD. Blood pressure values should be kept ≤120/80 mmHg, adapting this target according to patient tolerance. If a CKD is present, systolic blood pressure should be kept <120 mmHg [205]. As patients with LN frequently have nocturnal hypertension and reduced blood pressure dipping, 24-hour ambulatory blood pressure monitoring should be considered [269]. Non-dipper profile is associated with CKD progression and cardiovascular events [270]. Non-pharmacological measures in this setting include a low-sodium diet, physical activity (target of at least 150 min per week), and maintenance of ideal weight. Patient education plays an important role in helping to achieve these goals [271], as discussed in Section 7.3.

### 6.2. Control of Proteinuria

Reduction or prevention of proteinuria is in general a key measure to prevent or delay CKD progression. ACE inhibitors or sartans are first-line drugs not only to treat hypertension and prevent cardiovascular damage but also in case of isolated proteinuria [205,272]. SGLT2i may provide further nephro-cardioprotection in LN through their antiproteinuric effects, in addition to background therapy with ACE inhibitors or sartans, but their role in LN must still be defined [249], as previously discussed. Finerenone, a non-steroidal mineralocorticoid receptor antagonist, has also shown promising results in the treatment of persistent proteinuria despite therapy with ACE inhibitors or sartans in diabetic nephropathy and is being investigated in a phase III trial in CKD of non-diabetic aetiology [273].

### 6.3. Control of Dyslipidemia

Control of dyslipidemia through diet and lipid-lowering drugs is a key aspect and the target of LDL cholesterol reduction should be differentiated considering the degree of risk. According to the recommendations of the European Society of Cardiology [274], it should be <100 mg/dL for LN patients with normal renal function (moderate risk), <70 mg/dL in patients with CKD (high risk), and <50 mg/dL for those with clinical history or documented evidence of atherosclerotic cardiovascular disease (very high risk).

### 6.4. Prevention of Thromboembolism

Primary prophylaxis of CV events with low-dose aspirin (75–100 mg/d) may be considered on an individual basis in SLE patients with LN, whose cardiovascular risk is markedly higher than age and sex-matched healthy population and also compared to SLE without renal involvement; however, robust evidence supporting this approach is lacking [268]. Two settings which deserve special attention are presence of antiphosfolipid antibodies (aPL) and nephrotic syndrome. Primary prophylaxis with low-dose aspirin is recommended in the presence of aPL characterized by a high-risk profile and may be considered also in lower risk aPL patterns. In general warfarin is the gold-standard drug for secondary prophylaxis in case of previous events defining APS [275]. A more articulate therapeutic algorithm has been recently proposed by EULAR guidelines [276]. A recent systematic review showed that direct-acting oral anticoagulants rivaroxaban and apixaban were less effective than vitamin K antagonists in preventing thrombosis in patients with APS or positive for two or three different aPL types [277]. Another high-risk situation is nephrotic syndrome due to membranous LN, which requires starting prophylaxis with low molecular weight heparin or vitamin K antagonists in patients with an albumin concentration < 2.5 g/dL and associated risk factors (such as proteinuria >10 g/day), and continuing it until serum albumin reaches 3.0 g/dL [239]. Finally, a recent study has underlined the antiplatelet effect of HCQ by a total thrombus-formation analysis system [278], reinforcing the concept of a possible additive effect in association with low-dose aspirin [279].

### 6.5. Prevention of GC-Induced Osteoporosis

Incidence of fractures due to osteopenia ranges from 30% to 50% among patients on GC for more than three months [280]. Prevention of this complication includes minimization of GC cumulative dose [281] and correction of other modifiable risk factors, such as smoking, alcohol consumption and sedentarism [282]. An adequate amount of dietary calcium (1 g/day) and maintenance of satisfactory levels of vitamin D (>30 ng/mL) are important in this setting. Very high-risk patients (e.g., use of PDN equivalent ≥30 mg/day for more than 1 month; densitometric evidence of osteoporosis, age older than 40) should be treated with drugs including oral bisphosphonates, denosumab, teriparatide, and romosozumab [281].

### 6.6. Prevention of Infections

Infections are an important cause of mortality and morbidity in SLE patients, who are often treated with lifelong IS therapy [283]. A recent review has analyzed the topic of vaccines and antimicrobial prophylaxis in immunocompromised patients with kidney diseases [284]. Recommended or suggested vaccinations and prophylactic treatments to prevent reactivation of different infectious agents in SLE are outlined in Table 4. Vaccine responses are generally impaired, especially after the recent administration of B-cell depleting agents and high-dose MMF.

## 7. The Role of Non-Pharmacological Management

Beyond IS therapy and adjuvant therapies, the concept of wider, holistic and multidisciplinary management of SLE patients should be emphasized. Many non-pharmacological interventions are an essential complement to traditional therapy and may have an impact also on kidney disease.

### 7.1. General Lifestyle Measures

Recently published EULAR recommendations for non-pharmacological management of SLE encompass a wide range of tailored measures aimed at improving health-related quality of life and enhancing disease awareness and self-management [271]. Among the main objectives are cessation of smoking habit, avoidance of cold exposure to prevent Raynaud’s phenomenon, photoprotection to prevent flares, physical exercise to progressively increase aerobic capacity and potentially reduce fatigue, and psychosocial interventions to mitigate anxiety and depressive symptoms.

### 7.2. Dietary and Nutritional Aspects

Information and support about diet is another crucial aspect. A patient-centered nutrition counseling appears to impact weight control, intake of sodium, and percentage of calories from fat and saturated fat. It also favors an increase in fruits and vegetables, helping to achieve a high-fiber, low-cholesterol diet [285]. A recent systematic review has confirmed that a low-fat intake and Mediterranean diet may reduce cardiovascular risk, but large interventional studies are needed [286]. Diet appears to exert its beneficial effects also by modulating autoimmunity through gut microbiota (as discussed in Section 3.2) and probably also has a direct impact on renal inflammation [53]. Interestingly, a high-fat diet determined increases in germinal center B cells, TFH in the spleen, more severe renal lesions and proteinuria in MRL/lpr mice [287].

A low-protein diet could be considered in CKD as early as stage III (GFR < 60 mL/min) to delay the progression of chronic kidney damage and a controlled sodium intake is an important aid in managing arterial hypertension, which is very frequent in proliferative forms of LN [288]. Interestingly, dietary fibers can increase gut bacterial diversity and reduce inflammation in rheumatoid arthritis and other autoimmune disorders [289]. Supplementation of vitamin D, vitamin E, and omega-3 fatty acids appears to be effective in reducing inflammatory markers, potentially improving endothelial function [55]. Correcting vitamin D deficiency, which is common in SLE, may help mitigate disease severity and reduce steroid dose through its immunomodulatory effects on innate and adaptive immunity. Direct supplementation of active vitamin 1,25-dihydroxyvitamin D3 (calcitriol) should be considered if CKD is present [290].

Finally, significant deficiency in several micronutrient intakes has been reported in SLE patients and may require supplementation, but further studies are needed to clarify this aspect [291].

### 7.3. Patient Education and Support

The need for long-term treatment with side effects and the burden of chronic disease itself can significantly reduce adherence to medical advice and therapy, especially in adolescents and early adults, making communication a crucial aspect [292]. Behavioral and psychosocial interventions can have an impact on fatigue, anxiety, and depression, thus improving quality of life and compliance [293]. The coexistence of renal involvement makes these aspects even more relevant. Patients should be informed about the prognosis of their nephropathy and warned about the risks of taking nephrotoxic drugs such as non-steroidal anti-inflammatory agents [294] and over-the-counter medications [295]. Adequate counseling about contraception and pregnancy should be offered, emphasizing the importance of starting pregnancy in a quiescent phase and estimating additional risks derived from renal disease, especially if CKD or severe hypertension are present [296]. All these issues can be dealt with more easily with a high-quality patient-doctor relationship, based on mutual trust and shared decision-making [297]. This might also help reduce disparities in SLE survival rates due to race, ethnicity, and social disadvantage, which are still reported in many countries [13].

## 8. Current Limitations and New Perspectives

Although the prognosis of LN has improved over the last few decades, it remains an important cause of morbidity and mortality in SLE. Around 70% of patients do not achieve CR after a 6-month standard induction treatment and 10–30% of them still progress to ESRD within 10 years of diagnosis, with an associated burden of increased cardiovascular and infectious risk [298]. Prevention of relapses and preservation of renal function remain a challenge, as well as minimization of drug toxicity, primarily due to long-term GC therapy that compromises the prognosis of LN, with increasing risk of infections and deaths [192,193,299].

VOC and BEL improved clinical results when used early as induction agents on top of standard therapy [300] and allowed a reduction in the cumulative dose of GC which may significantly reduce long-term side effects. Still, also VOC and BEL are not devoid from Adverse Events (AEs) and limitations. In particular, VOC can worsen hypertension and reduce GFR, limiting its use in CKD, and long-term histological data on nephrotoxicity are lacking [220,221]. BEL is generally well tolerated, but an increased rate of psychiatric AEs such as insomnia, anxiety, and depression-related symptoms was observed [218].

In addition to these aspects, there is an unmet need for biomarkers to identify high-risk patients who require a stronger induction therapy with a triple-drug regimen to achieve CR and distinguish them from an important proportion who may be instead overtreated with this approach [257]. Other current limitations are the lack of definite criteria for GC-reducing protocols, duration of maintenance therapy, and safe withdrawal of IS drugs in quiescent disease; better management of these aspects would likely help reduce the burden of long-term drug AEs [239].

Other biologic agents such as new generation anti-CD20 (obinutuzumab), IFN-I antagonists (anifrolumab), and RTX-BEL sequential therapy or combination hold promise in improving the efficacy-to-toxicity balance in LN treatment and in offering new tools to manage refractory and relapsing forms, which represent a therapeutic challenge due to lack of evidence-based therapies [301]. Both drugs show promising AE profiles, considering that obinutuzumab and anifrolumab were not associated with increases in serious AEs, infections, or deaths [222,223,244].

In addition to these drugs, several new developments are paving the way for potential major progress in LN management. A new frontier is represented by engineered T cells which express chimeric auto-antibody receptors (DNA-CAART) and can selectively target B cells expressing anti-dsDNA autoantibodies. This therapy has been assessed also in an organoid model of LN in vitro, showing anti-apoptotic and anti-inflammatory effects which resulted in improvements in morphology [302]. Another expanding area of research is that of therapeutic manipulation of different ncRNAs, which could inhibit the expression of crucial genes involved in LN, as summarized in Table 5. This intervention could modulate multiple pathways and interfere with key immunological and inflammatory mechanisms of kidney damage [46].

An interesting area of research connected with the previous one concerns extracellular vesicles (EVs), which may play multiple pathogenic roles in SLE [308]. They can carry autoantigens or complement factors, promote IC deposition on the glomerular basement membrane, and trigger inflammatory responses and coagulation cascade [309]. Targeting surface molecules on EVs has been proposed to “kill the messenger” and thus blunt autoantigenic fueling of ICs and stimulation of adaptive immune cells [310]. EVs also carry a variety of bioactive molecules and genetic material, including miRs and lncRNAs, and transfer them to target cells mediating deleterious effects. For example, miR-181d-5p, which is increased within EVs derived from M0 macrophages in LN, can target human MC stimulating proliferation and pyroptosis by binding to BCL-2. Of interest, these effects were reversed when levels of miR-181d-5p in EVs were reduced, confirming the therapeutic potential of miRNA antagonism [311]. On the other hand, EVs released from mesenchymal stromal cells (MSCs) show immunosuppressive and anti-inflammatory properties and have been successfully employed in several autoimmune disorders and GNs, including SLE and LN [312]. The beneficial actions of EVs are largely mediated by the transfer of miRs and lncRNAs which target cells and modulate sets of critical genes [313]. Nanomedicine techniques are allowing interesting manipulations of EVs. Engineered EVs presenting CD40 on their membrane can disrupt the CD40/CD40 ligand (CD40L) costimulatory axis through a blockade of CD40L on CD4^+^ T cells and appear to blunt the production of antibodies from B cells and restrain the generation of germinal centers in MRL/lpr mice. Furthermore, MMF can be also encapsulated within EV to inhibit the activity of lymphocytes and DCs [314]. Cationic liposomes modified with a cell-penetrating peptide were able to carry a small interfering RNA (siRNA) which suppressed B cell proliferation through the TLR4 signaling pathway, resulting in reduced proteinuria and serum anti-dsDNA titer [315].

All these novel tools, however, would require an accurate selection of candidate patients to maximize their effectiveness. One of the obstacles that still remains is the etiopathogenetic heterogeneity of LN and its need for a better disease classification. Response to anti-CD20 monoclonal antibodies, for example, can be highly variable and predictive factors to guide this therapy are lacking [316]. Molecular characterization, gene-signature fingerprints, and omic panels might improve patient stratification and facilitate individualized treatment with targeted drugs in the near future [317]. Artificial intelligence and machine learning techniques have been providing new tools for the prediction of renal flare risk, which may be useful for clinical decision-making [318]. Furthermore, they are allowing important progress in defining the genetic landscape of LN. In a recent study, four hub genes (out of 270 differentially expressed genes) involved in immune cell infiltration were identified, namely *CD53*, *TGFBI*, *MS4A6A*, and *HERC6.* They are especially expressed in macrophages and correlate with histological class, renal function, and proteinuria [137]. Another recent study identified four hub genes (*STAT1*, *PRODH*, *TXN2*, and *SETX*), associated with oxidative stress related to LN and most correlated with activated B and CD8 T cells [138]. Transcript analysis of serial LN kidney biopsies demonstrated the evolution of gene expression in the kidney according to clinical response to therapy. This molecular landscape differentiating responders from non-responders may help guide LN treatment on the basis of the actual pathogenesis of kidney injury [139].

Overall, this progress may lead to a molecular diagnosis of LN based on genetic fingerprints and specific pathway activation. The wealth of information generated by “omic” approaches, integrated with artificial intelligence and machine learning tools, is likely to lead to the discovery of many new non-invasive biomarkers, which could replace traditional ones and potentially even renal biopsy [319].

## 9. Conclusions

Significant progress has been made in unraveling pathogenetic mechanisms of LN over the last years, with new insight into mechanisms triggering the production of autoantibodies and interactions between infiltrating immune cells and kidney resident cells. This has been accompanied by a progressive increase in the number of candidate serum and urine biomarkers, which may integrate information from renal biopsy and change its role in the near future. Also IS therapy has been significantly expanding, with new approved drugs, such as BEL and VOC, allowing a stronger induction and the possibility of modulating it according to clinical features. Many other drugs in the pipeline may become part of LN therapy over the next years.

This renaissance in LN management is raising hopes that currently unmet clinical needs will be satisfied more easily in the near future. Achieving a higher rate of CR, preventing flares and progression of LN, reducing drug toxicity and personalizing treatment may have an impact on long-term outcomes of this challenging disease.

## Figures and Tables

**Table 1:** Prevalence of Lupus Nephritis (LN) in renal biopsy registries from different countries.

Country	LN/All Diagnoses	Reference
USA	7%	[20]
Norway	4.1%	[21]
Belgium	4.1%	[22]
Germany	5%	[23]
Spain	8.7%	[24]
China	7.5%	[25]
Japan	6.5%	[26]
India	9.4%	[27]
Colombia	17.8%	[28]
Senegal	13.7%	[29]

**Table 3:** Main recent phase III trials in LN therapy.

Study (Publication Year)	Drug	Main Results	Other Results and Comments	References
BLISS LN (2020)	BEL	43% vs. 32% in CRR trial vs. SOC (*p* < 0.01)	Reduced incidence of renal and extra-renal flares and of renal events or death. It can be used in renal failure and during pregnancy. Impaired response if proteinuria > 3 g/d. It can be associated with either CYC or MMF and GC (triple induction therapy).	[218]
BEAT LUPUS (2021)	RTX-BEL sequential therapy	Reduced incidence of post-RTX flares	BEL maintenance therapy after RTX may be necessary to prevent flares in SLE refractory to conventional therapy.	[219]
AURORA 1 (2021)	VOC	41% vs. 23% in CRR trial vs. SOC (*p* < 0.01)	Rapid decrease in proteinuria (50% in 1 month). Aggressive GC tapering (PDN 2.5 mg/d by 16 weeks) successful in 80% of patients. It can be associated with MMF and GC as triple induction therapy.	[220]
AURORA 2 (2022–2023)	VOC	Stable GRF and no evidence of chronic nephrotoxicity at renal biopsy after 3 years.	Less nephrotoxicity than CsA and TAC, but requires GFR > 45 mL/min.	[221]
TULIP-LN (2022) and extension (2023)	Anifrolumab	27.3% vs. 17.8% CRR in intensive-regimen Anifrolumab vs. placebo at 2 years.	Improvement of GRF numerically higher in both intensive and basic regimen of Anifrolumab. Higher incidence of Varicella Zoster.	[222]
NOBILITY (2022)	Obinutuzumab	Primary endpoint not met; however, 38% vs. 16% in CRR trial vs. SOC at 76 week (*p* < 0.01) with PDN <7.5 mg/d and improved renal responses through week 104.	Superior preservation of renal function and prevention of LN flares at post-hoc analysis.	[223]

BEL—belimumab; CYC—cyclophosphamide; CRR—complete renal remission; GC—glucocortidoid; GFR—glomerular filtration rate; LN—lupus nephritis; MMF—mycophenolate mofetil; PDN—prednisone; RTX—Rituximab; SOC—standard of care; TAC—tacrolimus; VOC—voclosporin.

**Table 4:** Vaccinations and prophylactic treatments in SLE [283,284].

Vaccinations	Indications and Comments
Pneumococcus, meningococcus, haemophilus Influenzae B	Recommended in all SLE patients.
Tetanus, diphtheria, pertussis	
Influenza, hepatitis A and B	
Recombinant herpes zoster	Reactivation of herpes zoster virus is frequent in SLE patients and vaccination can be considered on an individual basis.
Papillomavirus	Risk factor for cervical cancer in SLE patients.
COVID-19	
**Prophylactic treatments**	
Hepatitis B and hepatitis C	Patients with chronic or past infection planned to be treated with lymphocyte depleting agents or intense IS.
Strongyloides stercoralis	Prophylaxis could be considered in patients planned to be treated with high dose GC pulses to prevent Strongyloides hyperinfection syndrome.
Pneumocystis jirovecii	Although debated, prophylaxis could be considered in patients planned to be treated with RTX or intense IS; presence of LN and lymphopenia (<500 lymphocytes/mm^3^) are among risk factors for reactivation.
Latent tuberculosis	Patients with positive epidemiology and/or positive screening test and/or radiological evidence of previous exposure.
Measles and chickenpox	Patients exposed to infected people in a contagious phase might be treated with hyperimmune immunoglobulin and varicella zoster immunoglobulin, respectively.

Legend: RTX—Rituximab, IS—immunosuppression.

**Table 5:** Potential therapeutic application of ncRNA in LN.

Type of ncRNA	Mechanism of Action	Comments	Reference
miR-410	Inhibition of IL-6 expression	Decreased IL-6, TFG-β1 and collagen I/III production by MC in experimental models.	[303]
miR-125a-3p	Inhibition of IL-17 and TGF-β1 expression	Suppression of fibrosis in LN mice.	[304]
miR-127-3p	Inhibition of IFN-I/JACK1 signaling	miR-127b-3p is a negative regulator of STING/IRF3/IFN-I signaling in MC and its expression is reduced in LN; miR-127-3p mimics could correct overactivation of IFN-I/JACK1 signaling in LN.	[305]
miR-145	Inhibition of CSF-1 expression	Down-regulation of CSF-1 inhibits JAK/STAT signaling pathway and LN development in vivo.	[306]
Knockdown of lncRNA RP11-2B6.2	Inhibition of IFN-I signaling	lncRNA RP11-2B6.2 expression is elevated in renal tissue in LN and correlates with expression of IFN-stimulated genes.	[307]
Knockdown of circRNA ELK4	Inhibition of miR-27b-3p	circRNA ELK4 correlates with LN activity. circELK4/ miR-27b-3p/STING/IRF3/IRN-I axis probably crucial in LN.	[305]
LNA-anti-miR-150	Inhibition of miR-150	miR-150 promotes MC aging and expression of pro-fibrotic genes; it is involved in M1 macrophage polarization.	[307]

Legend. miR—microRNA; IL—Interleukin; lncRNA—long non-coding RNA; circRNA—circular RNA; LNA—locked nucleic acid; MC—mesangial cell; IFN-I—type I interferon.

## Data Availability

The data employed for conducting this review are available upon request to the following e-mails: marco.quaglia@med.uniupo.it.

## Abbreviations

aCL	anticardiolipin antibody
ALCAM	activated leukocyte CAM
APC	antigen presenting cell
aPL	anti-phospholipid
APS	anti-phospholipid syndrome
APRIL	a proliferation-inducing ligand
BAFF	B cell activating factor for TNF family
BEL	belimumab
CAM	cell-adhesion molecule
circRNA	circular RNA
CKD	chronic kidney disease
CYC	cyclophosphamide
CNI	calcineurin inhibitor
CR	complete remission
CRR	complete renal remission
DC	dendritic cell
DEG	differentially expressed genes
ELNT	Euro Lupus Nephritis Trial
ESRD	end stage renal disease
EV	extracellular vesicle
Fn14	fibroblast growth factor-inducible 14
GC	glucocorticoid
GEC	glomerular endothelial cell
GN	glomerulonephritis
HCQ	hydroxychloroquine
HE4	human epididymis protein 4
IC	immune complex
IFN	interferon
IFN-I	type I interferon
IL	interleukin
IS	immunosuppressive
JAK	Janus Kinase
KIM1	kidney injury molecule 1
LNA	locked nucleic acid
lncRNA	long non-coding RNA
MC	mesangial cell
MCP-1	monocyte chemoattractant protein1
MERTK	MER tyrosine kinase
MHC	major histocompatibility complex
miR	microRNA
MMF	mycophenolate mofetil
MN	Monocyte neutrophil
MP	methylprednisolone
MPA	mycophenolic acid
mTOR	mammalian target of rapamycin
ncRNA	non-coding RNA
NET	neutrophil extracellular trap
NGAL	neutrophil gelatinase associated lipocalin
NK	natural killer
pDC	plasmacytoid dendritic cell
PDN	prednisone
PR	partial remission
RNP	ribonucleoprotein
RTX	rituximab
SGLT2i	sodium-glucose cotransporter-2 inhibitor
SLAMF	signaling lymphocyte activation molecule family
SLEDAI	systemic lupus erythematosus disease activity index
Sm	Smith
SOC	standard of care
STAT	signal transducer and activation of transcription
TEC	tubular epithelia cell
TFH	T follicular helper
TGFβ	transforming growth factor β
TMA	thrombotic microangiopathy
TNF	tumor necrosis factor
Th	T helper lymphocyte
Treg	T regulatory lymphocyte
TWEAK	TNF-like weak inducer of apoptosis
SAP	SLAM associated protein
SLAM	costimulatory lymphocyte associated molecule
SLE	systemic lupus erythematosus
VCAM-1	vascular CAM1
VOC	voclosporin
